# Pediatric Chronic Migraine Severity and Maternal Stress

**DOI:** 10.3390/pediatric13040068

**Published:** 2021-10-15

**Authors:** Daniela Smirni, Marco Carotenuto

**Affiliations:** 1 Department of Psychology, Educational Science and Human Movement, University of Palermo, 90128 Palermo, Italy; 2 Clinic of Child and Adolescent Neuropsychiatry, Department of Mental Health, Physical and Preventive Medicine, Università degli Studi della Campania “Luigi Vanvitelli”, 81100 Caserta, Italy; marco.carotenuto@unicampania.it

**Keywords:** primary headache, maternal stress, migraine without aura, PedMIDAS

## Abstract

Primary headache is an increasing phenomenon in pediatric age, and very often, it causes disabling limitations in children’s daily activities, negatively affecting family well-being. There are conflicting data in the literature on the impact of children’s migraines on parental experienced stress. This study aimed to evaluate maternal stress in a sample of school-aged children with a migraine without aura (MwoA) and its correlation with migraine intensity and frequency. A total of 474 mothers aged between 31 and 55 participated in the study: 237 were mothers of children with MwoA, and 237 were mothers of typical developing children. All participants were administered the Parent Stress Index-Short Form (PSI-SF) for the assessment of parental stress; the Pediatric Migraine Disability Assessment (PedMIDAS) was administered to children with MwoA to assess the presence of a related disability migraine. The results showed a significantly higher rate of stress in mothers of MwoA children (*p* < 0.001) in all the domains explored by the PSI-SF and a statistically significant correlation between the maternal stress total score and the intensity and frequency of migraine attacks (*p* < 0.0001). This study highlights the need for the holistic contribution of the family to be considered in the clinical management of pediatric migraines.

## 1. Introduction

A migraine is the most common cephalic pain in the developmental age [1], with the overall prevalence estimated to be about 9.1% (95% CI: 7.1–11.1) [2]. Chronic migraine is a disabling neurological disease with its relevant impact on quality of life negatively related to pain severity [3]. In fact, a recurring headache and the need for frequent medical examinations tend to negatively affect family well-being, and actually, up to 18% of admissions to the pediatric emergency department are due to primary headaches [4].

Moreover, in the developmental age, chronic migraine seems to often be associated with psychiatric, neurological and cardiac comorbidities [5]; learning impairment [6] and sleep problems [7,8,9,10]. 

To date, few studies have explored the impact of headache on parental stress with debating and conflicting results. Esposito et al. found that parents of children with a migraine without aura have higher stress levels than parents of healthy children [11]. On the contrary, Barone et al. and Operto et al. found no difference in the parenting stress of children with or without primary headaches [12,13]. 

An increasing number of studies have recently investigated how emotional/behavioral problems in children are associated with chronic disorders such as migraines; however, knowledge of the relationship between these problems and parental stress is still limited [14,15,16,17]. In general, parents of children with chronic medical, neurological or psychiatric conditions show elevations of perceived stress levels and a reduction of parental quality [18,19,20,21,22], and specifically, children with headaches seem to be at a high risk for psychological maladjustment, including internalizing disorders [23,24]. Within this perspective, the literature has reported how managing a problematic child can become a significant source of stress for parents, especially for the mother as the primary caregiver [21,25].

It is important to consider that parental stress can affect the child’s emotional disturbances, and this, in turn, can negatively affect the child’s pain perception [26]. Therefore, investigating the relationship between migraine in the child and primary caregiver stress is crucial for the proper management of this condition. 

However, some studies have also documented that a sympathetic progression and involvement of the autonomic system and the hypothalamus-pituitary-adrenal (HPA) axis could be at the basis of the neurobiological mechanism that favors migraine attacks and favors the development of chronic migraines, amplifying maternal stress [27,28,29]. Moreover, we cannot also exclude the role of the orexin/serotoninergic pathway interaction, the consequent dysregulation of wake–sleep cycling and the pain perception in migraine subjects independently from age [30] and the mutual effect of parenting stress and pediatric sleep troubles in MwoA children [31].

Although pediatric primary headaches are today a hot research topic, only a few studies have focused on the correlation between migraine severity and the degree of parental stress, and their results have often been contrasting. Therefore, this study aimed to evaluate maternal stress in a sample of school-aged children with migraine without aura (MwoA) and its putative correlation with migraine intensity, frequency and disability severity.

## 2. Materials and Methods

### 2.1. Participants

A sample of 474 mothers aged between 31 and 55 years (mean age 44.5 ± 8.15 years) participated in the study. The participants were all Caucasians, native Italian speakers and from a common mid-level socioeconomic status. The mothers participating in the study were all married women with stable and non-separated relationships. 

The study sample was divided into two groups. The first group consisted of 237 mothers of children with MwoA (120 boys and 117 girls; mean age 8.01 ± 1.45 years), recruited as inpatients from 2003 until 2013 at the Clinic for Child and Adolescent Neuropsychiatry of the University of Campania “Luigi Vanvitelli”. The diagnosis of MwoA had been made by a trained and expert-in-the-field physician (M.C.), and it was revaluated according to the third edition of the International Classification of Headache Disorders (ICHD-3) [32].

The second group consisted of 237 mothers of typically developing children (TDC) without migraine (122 males and 115 females; mean age 7.94 ± 1.63 years) from the same geographic region as the mothers in the first group. The mothers of the TDC children were randomly recruited from the parents of children attending public schools in Campania as part of a study on the typical neurological development of school-age and adolescent children.

Inclusion criteria for eligibility in the study were to be mothers of children that were:

(1) Males and females aged 6 to 12 years (inclusive);
(2) Of a typical neurodevelopment or with a diagnosis of migraine without aura, as e-evaluated by the HIS ICHD-3 guidelines (1.1 or 1.2 according to ICHD-3).

For the latter, in addition, they had:

- a history of migraine without aura of at least 6 months;
- a history of 4–14 migraine headache days and with at least 4 migraine attacks per month;
- a Pediatric Migraine Disability Assessment Scale (PedMIDAS) score >20 and <140 at first visit.

Mothers were excluded from recruitment if their children had:

(1) a psychiatric disorder, as reviewed by the Diagnostic and Statistical Manual of Mental Disorders, 5th edition [33] (i.e., psychosis, bipolar disorder, major depression, generalized anxiety disorder, post-traumatic stress disorder);
(2) documented neurodevelopmental disorders (i.e., autism spectrum disorders, cerebral palsy or cognitive impairment, ADHD);
(3) active and/or significant risk of suicide;
(4) an acute, severe or unstable medical condition.

### 2.2. Parenting Stress Index-Short Form (PSI-SF) 

To assess parental stress, the Italian version of the PSI-SF was administered to all participants [11,13,25,34,35]. 

The PSI-SF is a standardized self-administration questionnaire that provides scores on three different scales: (1) Parental Distress; (2) parent–child interaction; (3) difficult child.

The Parental Distress subscale evaluates the parent’s stress, per se. The parent–child interaction subscale evaluates the stress caused by the parent–child interaction. The difficult child subscale measures the stress of managing a child who appears more problematic than the parent expected.

The PSI-SF has 36 elements, based on a five-point Likert scale (1 = completely disagree; 5 = completely agree). The subscale scores range from 12 to 60, and the Total Stress score ranges from 36 to 180. The higher the score is, the greater the parental stress level [11,13,25,34,35].

The PSI-SF showed high internal consistency (Cronbach’s alpha: 0.92), and its reliability and validity have been widely reported, even in parents of children with chronic conditions. In the present study, the three subscales had adequate internal reliability (Cronbach’s alpha ranging from 0.57 to 0.70) [11,13,25,34,35].

### 2.3. Pediatric Migraine Disability Assessment (PedMIDAS) 

The Pediatric Migraine Disability Assessment (PedMIDAS) was completed by the child, the mother or both together. It is the only validated tool for assessing migraine disability among school-aged children. This tool consists of six questions that investigate the impact of migraines on daily activities and overall quality of life. The PedMIDAS has been validated and proven reliable for assessing migraine-related disability in children and adolescents. It is also useful for evaluating the results of interventions and comparing responses to different treatments [36].

*Ethical Approval*

The present study was conducted according to the principles of the Declaration of Helsinki [37]. The Ethics Committee at the Università degli Studi della Campania “Luigi Vanvitelli” approved the retrospective study design and all procedures, considering the adherence with international guidelines (Protocol code 0022404/i; 23 July 2021). Informed consent was obtained from all subjects involved in the study.

*Statistical Analysis*

The two groups were compared for demographic characteristics using an independent *t*-test. The independent *t*-test was also performed to compare the PSI-SF performance of the mothers of the two groups (MwoA vs. TDC). In addition, correlational analyses were carried out to verify if there was a relationship between the frequency, intensity and duration of migraine episodes and the level of parental stress. For all analyses, *p* values ≤0.05 were considered as statistically significant. The software Statistica version 8.1 (StatSoft Inc., Tulsa, OK, USA) was used for all statistical tests.

## 3. Results

No differences in age (*t*-test_(472)_ = 0.53; *p* = 0.59) nor in education (*t*-test_(472)_ = 1.52; *p* = 0.13) were found between the two groups.

As expected, comparing the stress levels of mothers of children with migraine problems and mothers of TDC, the mean comparisons of all parameters measured by the stress scale were statistically significant and had a very large effect size (Table 1). According to Sawilowsky’s “New Effect Size Rules of Thumb”, a Cohen’s d over 2 indicates a huge effect size [38]. 

Furthermore, the correlational analyses revealed positive and significant correlations between maternal stress levels and the intensity (r = 0.40; *p* < 0.0001) and frequency (r = 0.54, *p* < 0.0001), but not duration (r = 0.07; *p* = 0.253), of MwoA attacks (Table 2).

## 4. Discussion

The findings of the present study highlighted a suggestive relationship between the subjectively perceived maternal stress levels and the MwoA frequency, intensity and disability.

The clinical management of migraines in the developmental age is complex and also involves also the whole family. In 2013, Esposito et al. reported PSI-SF data from 218 children with MwoA and compared them to a large group of typically developing children, showing that parents of children with MwoA had significantly higher Parental Distress scores on the Parent–Child Interaction subscale, in the Difficult Child subscale and in Total Stress Score [11].

On the contrary, in 2015, Barone et al. compared the parental stress of 71 school-aged children with a headache (mean age 9.8 ± 1.3 years) and 71 typical developing children (mean age 9.2 ± 1 years), highlighting no difference between groups for parenting stress, using the PSI-SF to investigate stress perceived [12].

Similarly, in 2018, Operto et al., assessing 35 adolescents (mean age 14.89 ± 3.2 years) with migraine and 23 healthy adolescents (mean age 14.75 ± 3.17 years) through the PSI-SF, found no difference in terms of parenting stress (*p* = 0.29). However, interestingly, they found more internalizing problems (*p* = 0.023), affective problems (*p* < 0.01), anxious (*p* < 0.001), and somatic complaints (*p* < 0.001) among the headache group compared to control group [13]. 

On the other hand, the cited reports assessed only the perceived parental stress levels among the mothers of children affected by MwoA, without evaluating the possible correlation with the attacks intensity and frequency and not even with the disability degree. In fact, these are relevant parameters in clinical practice to calibrate the degree of severity of the individual patient and the effectiveness of the therapy.

The results of the present study showed a significantly higher rate of stress in mothers of children with MwoA (*p* < 0.0001), with a huge size effect in all the domains explored by the PSI-SF. Particularly, the chronical pain condition may contribute to create a false image of one’s child who only appears sick, and therefore, it is difficult to interact with him or her. Above all, it must be considered that any chronic pathology in pediatric age tends to alter the parental quality itself, with feelings of inadequacy and guilt on the part of the parents [20]. In this light, we can explain the perception of one’s child as difficult (Difficult child domain; *p* < 0.0001) as suffering from a chronic and disabling disease, such as MwoA at high frequency. However, it should be emphasized that this sort of rejection is due to the child’s pathology, a situation that generates feelings of helplessness in the parent (Parent–Child Interaction domain, *p* < 0.0001).

Moreover, this data can be considered as a relevant suggestion about the importance of coping strategies in chronic diseases, considering the significant negative impact on children’s daily lives and their families [39,40]. This may explain the higher values in the Parent–child interaction and Difficult child subscales among mothers of children with MwoA (*p* < 0.0001). 

Furthermore, the novelty of the present study can be identified in the correlational analyses between maternal stress levels and the frequency, intensity and duration of MwoA attacks. These analyses revealed that the degree of headache-associated disability (PedMIDAS) was strongly linked to the parent’s total stress level. On the other hand, the duration of migraine episodes was not related to the parent’s stress level.

It is conceivable that maternal stress, in turn, can increase the stress of the child, affecting the severity of the migraine. It is now widely recognized that chronic stress also is related to headache in developmental age [41,42], and in general, migraine attacks can be explained as a genetically determined adaptive behavioral response to stressors, internal or external, of a brain that feels threatened [43].

The neurobiological mechanisms by which stress could trigger migraine attacks and promote the development of a chronic migraine are unclear, although the dysfunction of the autonomic system and the hypothalamic-pituitary-adrenal (HPA) axis may be involved. Supportive of the presence of sympathetic dysfunction, serotonin and norepinephrine levels have been demonstrated to be lower in both those with PTSD and those with migraines [27,30,44]. 

Neurophysiological studies have shown abnormal cortical excitability and information processing in patients with migraines. It is possible that stress can produce neurochemical changes in the cerebral cortex, promoting migraine attacks [28].

In this scenario, Silvestro et al. showed that the imbalance between the need of investing resources to promote cerebral network efficiency and the need of minimizing the metabolic cost of wiring probably represents the mechanism underlying migraine patients’ susceptibility to triggers [45].

In general, we can consider the correlation between frequency, intensity and disability degree and maternal perceived stress as relevant. In fact, each chronic disease impacts the quality of life of the family and children affected, causing a stressful condition in pediatric age, with psychological troubles also in intra-familiar relationships [46]. Moreover, we can also speculate that frequent specialist clinical visits, the consequent perception of the sickness status and the effect of denial and rejection by parents of their child’s pathology may be the reasons underlying the high maternal stress related to the clinical characteristics of MwoA.

Therefore, children with MwoA may be more likely to benefit from behavioral interventions to improve stress assessment and coping strategies for treatment.

The current study’s main limitation is the lack of information on the stress level of fathers. As in a previous study on stress in parents of children with enuretic problems, it would be interesting to observe any differences in stress levels between mothers and fathers [21]. The choice to analyze only mothers’ stress levels arose from literature data which reported that mothers are more vulnerable to stress than fathers.

## Figures and Tables

**Table 1:** Mean comparison in the Parent Stress Index-Short Form (PSI-SF) between parents of children with migraine problems and typical developing children parents (TDC).

PSI-SF Scale	Migraine		TDC		t	*p*	Cohen’s d
	M	SD	M	SD	(df = 472)		
Parental distress	28.02	5.82	16.02	2.23	29.63	<0.0001	2.72
Parent–child interaction	32.44	5.20	15.67	2.37	45.18	<0.0001	4.15
Difficult child	31.23	5.62	16.77	1.67	37.99	<0.0001	3.49
Stress total score	91.69	11.75	48.47	5.24	51.69	<0.0001	4.75

PSI-SF: Parent Stress Index-Short Form; t: *t*-test; *p*: *p*-value; df: degrees of freedom; M: mean; SD: standard deviation.

**Table 2:** Correlation between frequency, duration, intensity and degree of headache-associated disability and parent’s total stress score.

	Total Stress Score	
	r	*p*
Frequency	0.54	<0.0001
Duration	0.07	0.253
Intensity	0.40	<0.0001
PedMIDAS	0.62	<0.0001

r: correlation’s coefficient; *p*: *p*-value; PedMIDAS: Pediatric Migraine Disability Assessment.

## Data Availability

Data supporting the reported results can be obtained on request.
